# Mutation analysis of the *AATF *gene in breast cancer families

**DOI:** 10.1186/1471-2407-9-457

**Published:** 2009-12-21

**Authors:** Maria Haanpää, Mervi Reiman, Jenni Nikkilä, Hannele Erkko, Katri Pylkäs, Robert Winqvist

**Affiliations:** 1 Laboratory of Cancer Genetics, Oulu University Hospital, P.O. Box 22, FIN-90221 Oulu, Finland; 2 Department of Clinical Genetics and Biocenter, University of Oulu, P.O. Box 5000, FIN-90014 University of Oulu

## Abstract

**Background:**

About 5-10% of breast cancer is due to inherited disease predisposition. Many previously identified susceptibility factors are involved in the maintenance of genomic integrity. AATF plays an important role in the regulation of gene transcription and cell proliferation. It induces apoptosis by associating with p53. The checkpoint kinases ATM/ATR and CHEK2 interact with and phosphorylate AATF, enhancing its accumulation and stability. Based on its biological function, and direct interaction with several known breast cancer risk factors, *AATF *is a good candidate gene for being involved in heritable cancer susceptibility.

**Methods:**

Here we have screened the entire coding region of *AATF *in affected index cases from 121 Finnish cancer families for germline defects, using conformation sensitive gel electrophoresis and direct sequencing.

**Results:**

Altogether seven different sequence changes were observed, one missense variant and six intronic ones. Based on the *in silico *analyses of these sequence alterations, as well as their occurrence in cases and controls, none of them, however, were predicted to be pathogenic.

**Conclusions:**

To our knowledge, this is the first study reporting the mutation screening of the *AATF *gene in familial breast cancer cases. No evidence for the association with breast cancer was observed.

## Background

In most Western populations, about one in ten women develop breast cancer [1]. Approximately 5-10% of these cases are considered to be familial [2]. Mutations in two major high penetrance genes *BRCA1 *and *BRCA2 *are well known, but they seem to be responsible for less than 20% of heritable disease predisposition [3,4]. Only a small number of the familial cases are explained by mutations in other known cancer susceptibility genes, such as *TP53, PTEN, ATM, CHEK2, NBS1, RAD50, BRIP1 *and *PALB2 *[5,6]. The identification of additional genes involved in breast cancer predisposition is complicated by genetic heterogeneity. The remaining cases could be the result of a few additional, yet unidentified, high penetrance mutations, but the polygenic model may provide a more plausible explanation [7]. Recent genome-wide association studies have identified a few common low penetrance breast cancer susceptibility alleles. Together these loci are, however, estimated to account less than 4% of the familial risk of breast cancer in European populations [1]. As most of the known breast cancer susceptibility genes are involved in DNA damage response pathways, other genes involved in these essential and highly complex and multilayered processes represent excellent candidates for identifying further cancer predisposing alleles.

**Figure 1:**
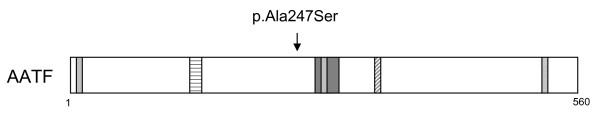
**Schematic diagram of AATF protein structure and location of observed missense variant**. Structure and main functional domains of AATF are shown. Leucine zipper motif location (aa 275-296) is marked in dark grey, with the basic domain in horizontal stripes (aa 133-145). Nuclear translocation signal is shown in diagonal stripes (aa 338-344). Three nuclear receptor binding motifs are marked in light grey (aa 11-15, 281-285, 520-524). The location of the observed amino acid alteration is shown by an arrow (functional domain structure modified mainly from reference [9]).

AATF (apoptosis antagonizing transcription factor, also know as CHE1) was originally characterized as an interacting protein for RNA polymerase II. The *AATF *gene is located at chromosome 17q11.2-q12. It encodes a phosphoprotein containing 558 amino acids [8] and consists of 12 exons. The protein is highly conserved among eukaryotic species during evolution [9]. AATF does not display homology to any previously described protein. It contains a leucine zipper motif, several phosphorylation sites for different kinases, a nuclear localization signal motif, three nuclear receptor LXXLL binding motifs and several four nucleotide repeats (Figure 1) [9,10]. The functions of AATF are essential during the early stages of embryogenesis and cell proliferation [11,12]. One significant function of AATF is to promote cellular transcription, acting as an adaptor that links specific transcription factors to the general transcription apparatus. Furthermore, due to its interaction with various important components of the cell survival machinery, AATF has been found to play an important role in DNA damage response, cell-cycle checkpoint control, apoptosis and also in chromatin remodeling [13]. Interestingly, AATF operates in a dualistic way, showing both inhibitory and stimulatory roles in regard to cell-cycle progression and cell proliferation [10].

AATF is also a nuclear receptor co-activator and regulates the physiological effect of p53. The p53 protein plays a critical role in the cellular response to DNA damage and other stresses by inhibiting proliferation or by inducing apoptosis [14]. Upon DNA damage, AATF is phosporylated by ATM and CHEK2, consequently increasing its stability and accumulation to the cell nucleus, but also enhancing p53 expression and G2-M arrest [10,15]. Although AATF induces apoptosis by associating with p53, it also has an antagonistic role in several cell types, acting as an inhibitor of apoptosis [10]. Its function as transcription factor or as co-activator has not yet been fully worked out. However, there seem to be different modes by which AATF cooperates with other transcription factors [16]. Based on all these observations it is reasonable to speculate that AATF may be a component of the checkpoint anticancer barrier that protects cells from DNA damage or oncogenic stress. Furthermore, AATF has been found to be down-regulated in several colon carcinomas and is involved in growth arrest through induction of p21 [17].

Based on its biological function, we wanted to determine whether *AATF *germline mutations are involved in hereditary susceptibility to breast cancer. We have, therefore, screened the entire coding sequence and exon-intron boundaries of the gene in Finnish cancer families. *AATF *sequence alterations have not previously been studied in relation to breast cancer predisposition.

## Methods

### Cases and controls

Breast and breast-ovarian cancer families (N = 121) originating in northern Finland were selected for the screening of possible germline mutations in *AATF*. Inclusion criteria for the 70 (58%) families classified as high-risk ones were the following: 1) three or more cases of breast, or breast and ovarian cancer in first- or second-degree relatives, or 2) two cases of breast, or breast and ovarian cancer in first- or second-degree relatives, of which at least one with early disease onset (≤ 35 years), bilateral disease or multiple primary tumors, including breast cancer in the same individual. Most of the high-risk families had three or more cases. The remaining 51 (42%) families with moderate disease susceptibility displayed two cases of breast cancer in first- or second-degree relatives. All high-risk families were previously screened for germline mutations in known or potential susceptibility genes *BRCA1, BRCA2, CHEK2, TP53, RAD50, RAP80 *or *PALB2 *[6,18-22], and disease associated alterations in these genes were seen in altogether 17 of the families. These mutation-positive families were included in the current study because we did not want to rule out potential genetic modifier effects. The frequency of all observed germline variants were determined in control samples (N ≥ 307) obtained from anonymous cancer-free female Finnish Red-Cross blood donors originating in the same geographical region as the studied families. All patients had given their informed consent for obtaining pedigree data and blood specimens for the study of cancer susceptibility gene mutations. An approval to perform the study was obtained from the Ethical Board of the Northern Ostrobothnia Health Care District and the Finnish Ministry of Social Affairs and Health.

### DNA isolation and mutation analysis

DNA was extracted from blood lymphocytes using the standard phenol-chloroform method or the Purgene D-50 K purification kit (Gentra, Minneapolis, USA). The entire coding region and exon-intron boundaries of the *AATF *gene were screened for germline mutations by conformation sensitive gel electrophoresis (CSGE) [23]. Samples with band shifts were reamplified and the sequencing analysis was performed on a Li-Cor IR^2 ^4200-S DNA Analysis system (Li-Cor Inc., Lincoln, USA) using the SequiTherm EXEL TM II DNA Sequencing Kit-LC (Epicentre Technologies, Madison, USA). Oligonucleotides for CSGE and sequencing were designed using the Primer3 software based on sequence information obtained from public databases (Genomic sequence NC_000017.9, mRNA NM_012138.3). Primers and PCR conditions for mutation screening and sequencing are available upon request.

### Statistical and bioinformatic methods

Carrier frequencies were compared by using Pearson's χ^2 ^or Fisher's exact test with SPSS version 16.0 for Windows (SPSS Inc., Chicago, USA). All P-values were two-sided. The missense alteration c.739G>T (p.Ala247Ser) was tested for possible pathogenicity by using PolyPhen software. ESEfinder software was applied to determine if the exonic variant was located in an ESE (exonic splicing enchancer) sequence and might, therefore, affect the ESE function. All alterations were also checked for potential splicing effects with NNSplice software.

## Results

The study of 121 breast or breast-ovarian cancer families revealed altogether seven different germline changes in the *AATF *gene (Table 1). Only one of the observed changes was exonic. This novel alteration resulted in an Ala247Ser amino acid substitution in the protein product. All the other seen variants were intronic. In order to evaluate possible pathogenicity of the observed changes, their frequencies were compared between cases and healthy control individuals. Assessment of the consequences of the observed changes was also done by using PolyPhen, ESEfinder and NNSplice software.

**Table 1:** Observed germline alterations in the *AATF *gene in Finnish breast cancer families

			Carrier frequency^a ^(%)			
Exon/Intron	Nucleotide change	Effect on protein	Familial cases	Controls	P-value (OR, 95% CI)	Previously known (+) or unknown (-) alteration
Ex 4	c.739G>T	p.Ala247Ser	1.7 (2/121)	1.3 (4/317)	0.70 (1.3, 0.2-7.1)	-
Int 3	c.694+35A>C	None	4.1 (5/121)	9.1 (30/328)	0.08 (0.4, 0.2-1.1)	+; RS: 8067751
	c.694+48T>G	None	3.3 (4/121)	0.9 (3/328)	0.09 (3.7, 0.8-16.8)	-
Int 4	c.832+17C>T	None	0.8 (1/121)	0.3 (1/317)	0.48 (2.6, 0.16-42.4)	-
	c.832+39C>T	None	0.8 (1/121)	- (0/317)	0.28 (NA)	-
Int 11	c.1619+29A>C	None	2.5 (3/121)	0.3 (1/325)	0.06 (8.2, 0.8-80.0)	-
Int 12	c.1670+42C>T	None	20.7 (25/121)	23.5 (72/307)	0.53 (0.9, 0.5-1.4)	+; RS:11653434

NA, not available; OR, odds ratio; CI, confidence interval; ^a^Heterozygotes

The p.Ala247Ser alteration was observed in 1.7% (2/121) of the patients and 1.3% (4/317) of the controls (P = 0.7). These two amino acids display very different characteristics. Alanine is a small hydrophobic and aliphatic amino acid, whereas serine is a polaric and hydrophilic residue. Based on the analysis using PolyPhen software the effect of this change, however, was predicted to be neutral. Neither had any influence on splicing nor ESE functions indicated.

All six intronic alterations observed were single nucleotide changes, four of which were novel ones, whereas two had already been described earlier in the internet-based sequence variation database http://www.ncbi.nlm.nih.gov/projects/SNP/. Most of the alterations were present in cases and controls at similar frequencies. However, one of the variants, c.832+17C>T, was only found among cases 0.8% (1/123) but not in controls (0/317). Furthermore, one alteration, c.1619+29A>C, occurred more frequently among cases 2.5% (3/121) than in controls 0.3% (1/324), but nevertheless the difference was not statistically significant (P = 0.066). According to the analysis using NNSplice software, none of the intronic changes observed had any effect on splicing.

## Discussion

The aim of our study was to determine the relationship between breast cancer susceptibility and potential alterations in the *AATF *candidate gene, which plays an important role in the maintenance of genomic integrity and cell-cycle checkpoint control [10]. Because of its influence on vital cellular functions it was considered possible that mutations in the *AATF *gene might contribute to hereditary disposition to breast cancer.

In the current study, the whole coding region of the *AATF *gene was systematically screened for mutations in 121 breast cancer families. We found several sequence variants in the *AATF *gene: one exonic and six intronic ones. The observed exonic change c.739G>T (p.Ala247Ser) was a novel one, but it located outside the functionally important domains. Furthermore, none of the intronic changes seemed to affect consensus splicing sequences. All observed variants displayed similar allele frequencies in cases and controls. Consequently, none of the observed alterations seemed to associate with an increased cancer risk. The absence of deleterious germline mutations in the *AAFT *gene could indicate conserved and essential function of the protein in cell cycle control and DNA damage response. However, a small study like this cannot exclude the possibility of rare mutations in *AATF *that might predispose to breast cancer, but based on our findings, they unlikely make any sizeable contribution to cancer predisposition.

## Conclusions

The observed *AATF *gene alterations lacked association with breast cancer risk and therefore mutations in this gene are likely not to play a significant role in hereditary predisposition to this malignancy. To our knowledge, this is the first investigation reporting the mutation screening of the *AATF *gene in familial breast cancer cases.

## Competing interests

The authors declare that they have no competing interests.

## Authors' contributions

MH, MR and JN designed the oligonucleotide primers, and MH and MR conducted the laboratory work. MH carried out the *in silico *as well as statistical analysis of the obtained genetic data and drafted the manuscript. RW conceived the study, and participated in its design and coordination together with KP. All authors contributed to the preparing of the manuscript and also read and approved the final manuscript.

## Pre-publication history

The pre-publication history for this paper can be accessed here:

http://www.biomedcentral.com/1471-2407/9/457/prepub
